# Reliable resolution of ambiguous hepatitis C virus genotype 1 results with the Abbott HCV Genotype *Plus* RUO assay

**DOI:** 10.1038/s41598-019-40099-3

**Published:** 2019-03-06

**Authors:** V. Saludes, A. Antuori, B. Reinhardt, I. Viciana, E. Clavijo, L. Schreiber, M. Tenenbaum, F. Rodriguez-Frias, J. Quer, L. Matas, E. Martró

**Affiliations:** 1 Microbiology Department, Laboratori Clínic Metropolitana Nord, Hospital Universitari Germans Trias i Pujol; Genetics and Microbiology Department, Universitat Autònoma de Barcelona, Badalona, Spain; 2 Germans Trias i Pujol Research Institute (IGTP), Badalona, Spain; 3 Centro de Investigación Biomédica en Red en Epidemiología y Salud Pública (CIBERESP), Instituto de Salud Carlos III, Madrid, Spain; 4 Abbott GmbH & Co. KG, Wiesbaden, Germany; 5 Microbiology Service, Hospital Virgen de la Victoria, Málaga, Spain; 6 Maccabi Mega-Lab, Rehovot, Israel; 7 Liver Pathology Lab, Biochemistry and Microbiology Department, University Hospital Vall d’Hebron, Barcelona, Spain; 8 Centro de Investigación Biomédica en Red (CIBER) de Enfermedades Hepáticas y Digestivas (CIBERehd), Instituto de Salud Carlos III, Madrid, Spain; 9 Liver Unit, Liver Diseases-Viral Hepatitis, Vall d’Hebron Institute of Research (VHIR-HUVH), Barcelona, Spain

## Abstract

Accurate subtyping of hepatitis C virus genotype 1 (HCV-1) remains clinically and epidemiologically relevant. The Abbott HCV Genotype *Plus* RUO (GT *Plus*) assay, targeting the core region, was evaluated as a reflex test to resolve ambiguous HCV-1 results in a challenging sample collection. 198 HCV-1 specimens were analysed with GT *Plus* (38 specimens with and 160 without subtype assigned by the Abbott RealTi*me* Genotype II (GT II) assay targeting the 5’NC and NS5B regions). Sanger sequencing of the core and/or NS5B regions were performed in 127 specimens without subtype assignment by GT II, with “not detected” results by GT *Plus*, or with mixed genotypes/subtypes. The remaining GT *Plus* results were compared to LiPA 2.0 (*n* = 45) or just to GT II results if concordant (*n* = 26). GT *Plus* successfully assigned the subtype in 142/160 (88.8%) samples. “Not detected” results indicated other HCV-1 subtypes/genotypes or mismatches in the core region in subtype 1b. The subtyping concordance between GT *Plus* and either sequencing or LiPA was 98.6% (140/142). Therefore, combined use of GT II and GT *Plus* assays represents a reliable and simple approach which considerably reduced the number of ambiguous HCV-1 results and enabled a successful subtyping of 98.9% of all HCV-1 samples.

## Introduction

Chronic infection with hepatitis C virus (HCV) can progress to liver cirrhosis, hepatocellular cancer and death^1^. In 2015, 71 million people worldwide were estimated to be chronically infected^2^. Being highly genetically diverse, HCV has been classified into 8 major genotypes and 86 confirmed subtypes^3,4^. Genotypes 1, 2 and 3 are distributed worldwide, genotype 1 being the predominant one, specifically subtypes 1a and 1b^5^. In Spain, a 1.5% HCV seroprevalence has been estimated in the general population^6^, with genotype 1 being the most prevalent one (66.9%), with subtype 1b predominating over 1a^7^.

Since the efficacy and barrier to resistance of non-pangenotypic direct-acting antiviral agents (DAAs) depend on the HCV genotype and subtype (especially for subtype 1a and 1b), HCV genotyping must be performed prior to treatment initiation and will determine the choice of therapy^8^. Additionally, genotyping upon treatment failure may differentiate between relapse and reinfection^9^. Furthermore, knowledge of circulating genotypes and subtypes is necessary for epidemiological purposes^10^.

While commercial genotyping assays use sub-genomic regions such as core or NS5B in addition to the more conserved 5′ untranslated (5′NC) region, the high genetic variability and small differences between genotypes and subtypes still remain a challenge for both real-time PCR and line probe-based HCV genotyping assays. This also applies to HCV-1 subtyping^11–21^. In case of ambiguous results, the use of a second genotyping method may help guiding treatment selection. Nucleotide sequencing and phylogenetic analysis of the NS5B or core/E1 regions has been recommended as the reference genotyping method in consensus proposals^22^. However, even this method is not able to resolve every individual sample, e.g. due to failure of amplification^11,13,17,23^. Furthermore, the procedure is considered impractical for most clinical laboratories because it is time-consuming, less sensitive^21^, may be technically challenging, and does not readily allow for detecting mixed-type infections.

The Abbott RealTi*me* HCV Genotype II assay (GT II, Abbott Molecular Inc.) is a real-time PCR assay which includes specific probes for the identification of genotypes 1 to 6 (5′NC region), and subtypes 1a and 1b (NS5B region). Using this assay, about 5.4% of all genotypes 1 were not classified at the subtype level in our centre^14^. The novel HCV Genotype *Plus* RUO assay (GT *Plus*, Abbott Molecular Inc., Des Plaines, USA) has been designed to complement GT II or other assays as a reflex test in order to resolve ambiguous HCV-1 results. By targeting the core region, it identifies subtypes 1a, 1b and genotype 6.

The aims of this study were: (i) to evaluate the performance of the GT *Plus* assay in a challenging collection of genotype 1 clinical specimens not subtyped by the GT II assay and obtained from three geographical regions; and (ii) to assess its accuracy at identifying 1a and 1b subtypes in comparison with the reference method.

## Material and Methods

### Study design

**Figure 1:**
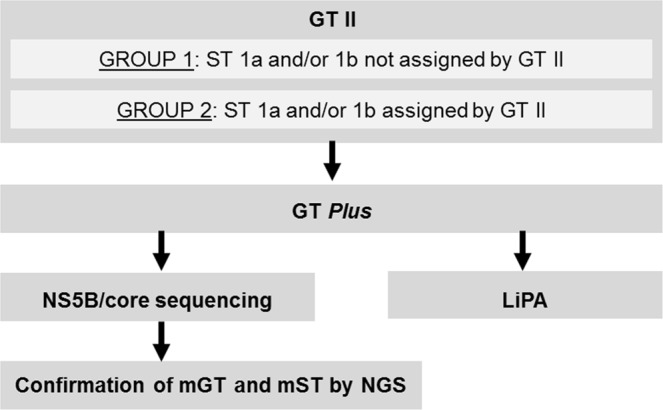
Flowchart diagram of the study design and methods used. GT, genotype; ST, subtype; GT II, Abbott RealTi*me* HCV Genotype II assay; GT *Plus*, Abbott HCV Genotype *Plus* RUO assay; LiPA, VERSANT HCV Genotype 2.0 assay; mGT, mixed genotypes; mST, mixed 1a and 1b subtypes; NGS, next-generation sequencing.

A flowchart diagram of the study design and the methods used is shown in Fig. 1. A total of 198 samples were included in this study and classified into two groups. Group 1 consisted of 160 genotype 1 samples for which no subtype had been assigned by the GT II assay upon routine testing (including eight samples with mixed genotypes). These samples were retrospectively collected in three different geographic areas in order to assess the performance of the GT *Plus* assay in subtyping these challenging samples. Group 2 consisted of 38 genotype 1 samples with subtypes assigned by the GT II assay (16 identified as 1a, 16 as 1b including three mixed genotypes, and six mixed 1a + 1b subtypes). The Group 2 samples were included in order to assess the concordance between both Abbott assays (GT II and GT *Plus*). Overall, the two groups included 17 mixed infections (11 mixed genotypes and six mixed subtypes).

All samples from Groups 1 and 2 underwent testing with the GT *Plus* assay. Subsequently, samples in Group 1 were subjected to the reference method for HCV genotyping based on Sanger sequencing and phylogenetic analysis of the targets of the two assays (NS5B region for GT II, and core region for GT *Plus*), except for 44 samples from Israel; GT *Plus* results for these 44 samples and another sample that failed Sanger sequencing were compared to the results of the line probe-based VERSANT HCV Genotype 2.0 Assay (LiPA, Siemens Healthcare GmbH, Erlangen, Germany) according to the manufacturer’s procedure. Out of Group 2, only samples with discordant results between the two Abbott assays, including partially discordant results in the case of mixed genotypes or subtypes, were subjected to the reference sequencing method. The result of one discordant sample from Israel was compared to LiPA. Finally, all samples with suspected mixed genotypes or subtypes or discordant results between core and NS5B sequences were further characterised by next-generation sequencing (NGS). The HCV genomic targets of the molecular techniques used in this study are listed in Table 1.

**Table 1:** Genotyping methods used and HCV genome target regions.

Genotyping method	Targeted genomic region and genotypes/subtypes detected		
	5′NC	Core	NS5B
GT II	1, 2, 3, 4, 5, 6	—	1a, 1b
GT *Plus*	—	1a, 1b, 6	—
LiPA	1, 2a/2c, 2b, 3, 4, 5a, 6	1a, 1b, 6c-6l	—
Sanger sequencing	—	Primers designed for genotype 1. Full region (nt 342–914)	Pangenotypic primers. Partial region (nt 8259–8639)
NS5B NGS	—	—	Pangenotypic primers. Partial region (nt 8254–8641)
5′NC-core NGS	Pangenotypic primers. Partial region (nt146-341)	Pangenotypic primers. Partial region (nt 342–914)	—

GT II, Abbott RealTi*me* HCV Genotype II assay (Abbott Molecular Inc.); GT *Plus*, HCV Genotype *Plus* RUO assay (Abbott Molecular Inc.); LiPA, VERSANT HCV Genotype 2.0 Assay (Siemens Healthcare GmbH); NC, non-coding; NGS, next-generation sequencing; nt, nucleotide.

This study was approved by the Ethics Committee (Comitè d'Ètica de la Investigació Clínica –CEIC–, Germans Trias i Pujol University Hospital, coordinator centre), and was conducted in agreement with the Declaration of Helsinki, Organic Law 15/1999 of 13 December about the protection of personal data, Law 14/2007 of 3 July on Biomedical Research, and Guideline for Good Clinical Practice of 2016. Informed consent was waived as anonymised retrospective samples were used.

### Clinical specimens

Left-over plasma or RNA samples archived at −80 °C were retrospectively selected from three laboratories in different geographic locations: (i) Germans Trias i Pujol University Hospital (Badalona, North-eastern Spain) (*n* = 74); (ii) Virgen de la Victoria University Hospital (Málaga, Southern Spain) (*n* = 64); and (iii) Maccabi Mega-Lab (Rehovot, Israel) (*n* = 60). These specimens were selected according to previous GT II assay results as follows: genotype 1 with no subtype assigned (Group 1, *n* = 160), or genotype 1 with subtype assigned (Group 2, *n* = 38) including 1a (*n* = 16), 1b (*n* = 16) and 1a + 1b (*n* = 6).

### Genotyping by Abbott GT II and GT *Plus* assays

Both genotyping assays are based on real-time PCR technology. Plasma specimens were tested with the fully automated Abbott *m*2000 system, including RNA extraction, PCR plate set-up, amplification and detection. When only previously extracted RNA was available, the amplification mix and PCR plate were prepared manually. The GT II assay identifies genotypes 1, 2, 3, 4, 5, and 6 with fluorescence-labelled oligonucleotide probes targeting the 5′NC region, as well as subtypes 1a and 1b targeting the NS5B region. When a fluorescent signal is only obtained from the HCV-1 specific probe (5′NC region) but 1a and 1b specific probes fail to provide a valid signal, HCV-1 subtype cannot be assigned. The GT *Plus* assay detects genotypes 1a, 1b, and 6 in a single reaction with probes targeting the core region. Results are interpreted by Abbott software as 1a, 1b, 6, or “not detected”. All analyses were performed following the manufacturer’s recommendations.

### Genotyping by NS5B and core sequencing and phylogenetic analysis

This method served as gold standard to assess (i) the accuracy of both Abbott genotyping assays in identifying 1a and 1b subtypes, and (ii) the presence of mismatches within the target regions for primers and probes of these assays. Starting from RNA extracted with the Abbott *m*2000*sp* system, retrotranscription reactions were performed in a 30 μl volume containing 7.5 μl eluted RNA, 0.25 mM dNTPs, 150 U Moloney murine leukaemia virus reverse transcriptase and 6 μl 5× RT buffer (Promega), 30 U RNasin RNase inhibitor (Promega), and 0.75 μg random hexadeoxynucleotides (Roche) in order to retrotranscribe the whole HCV genome preventing any bias during the reaction. The reactions were incubated at 42 °C for 2 min, 20 °C for 5 min, 25 °C for 5 min, 30 °C for 5 min, 35 °C for 5 min, 37 °C for 5 min, 42 °C for 45 min, and 2 min at 94 °C.

NS5B region was partially amplified and sequenced using a pangenotypic strategy as previously described^24^. To amplify the whole core region of HCV genotype 1 (573 bp, H77 positions 342–914), three different strategies were used. Supplementary Table S1 lists primers and thermal cycler profiles used in each PCR strategy. The first strategy was performed as previously published^25^. The second strategy consisted of two PCR rounds performed each in a 50 μl reaction volume with 5 µl cDNA, 5 μl *Pfu* DNA polymerase 10× buffer, 1.5 U *Pfu* DNA polymerase (Promega, Mannheim, Germany), 0.25 mM dNTPs, and 0.3 µM primers. The third amplification alternative consisted of two PCR rounds performed each with the amplification mix described above.

Amplification products were size selected and purified with Agencourt AMPure XP system (Beckman Coulter) following manufacturer’s instructions, and subjected to bidirectional Sanger sequencing with second round amplification primers. Sequence readings were assembled and edited with the STADEN package v1.6^26^. Study sequences were aligned by ClustalW implemented in MEGA 6^27^ together with reference sequences. Then, jModeltest^28^ was used to obtain the evolutionary model that best fitted the data according to the Akaike information criterion. This model was used to reconstruct a maximum likelihood phylogenetic tree with PHYML^29^. The robustness of the tree topologies was assessed by bootstrap analysis implemented in PHYML (500 replicates). Information regarding the presence of mutations in the primer/probe-binding regions of the commercial assays was kindly provided by Abbott as these sequences are proprietary.

### Genotyping with the VERSANT HCV Genotype 2.0 Assay (LiPA)

The LiPA assay is based on the reverse hybridisation of biotinylated amplified products from the 5′NC and core regions. The genotypes/subtypes 1, 1a, 1b, 1a/1b, 2a/2c, 2b, 3, 4, 5a, 6 and 6c-6l can be identified. Probes in the core region allow for a more reliable identification of subtypes 1a, 1b and 6c-l than the previous version of this assay. RNA extraction was performed using the Abbott *m*2000sp instrument and reagents. Hybridisation and detection steps were performed manually, following the manufacturer’s instructions.

### Assessment of mixed infections by NGS

NS5B amplicons, as well as 5′NC-core ones if necessary (spanning the first 130 bp of the core region), were subjected to NGS and subtype calling using the methodology and primers previously described^30^. Briefly, a RT-PCR-nested amplification was performed, and purified PCR products were quantified using the PicoGreen assay (Invitrogen, Carlsbad, CA, USA), and quality analysed using the BioAnalyzer DNA 1000 LabChip (Agilent, Santa Clara, CA, USA) prior to sequencing. PCR products from multiple patients individually identified by a genetic barcode were mixed at equal molecular proportion, and sequenced using MiSeq sequencing platform with MiSeq reagent kit v3 (Illumina, San Diego, CA). The raw data was filtered and processed to obtain a fasta file per sample which then was subjected to a phylogenetic analysis together with reference sequences. This multiple alignment provided a matrix of genetic distances with the Kimura-80 model with a gamma shape parameter of 0.42. The query haplotype was classified as the subtype with the nearest reference sequence. For subtypes with multiple reference sequences, two other distances to the query haplotype were computed: the average and the farthest in each subtype. These distances were used as clustering quality scores. A high-quality clustering result was produced when the subtyping according to the shortest, the average, and the farthest distances coincide. A confidence score was produced by 200 cycles of bootstrap analysis on the multiple alignment obtained for each query haplotype and subtyping according to the shortest distance. Query haplotypes classified with discordant shortest-distance and average-distance subtyping or with bootstrap scores below 70 were tagged as doubtful. A minimum of 5 reads and of 1% abundance was imposed to infer a mixed infection by a minority subtype. The data analysis system consisted of four R modules, implementing the demultiplexing module, the quality filter and haplotype selection module, the phylogenetic analysis module, and the expert system module.

## Results

All HCV genotype and subtype results according to the different genotyping methods used are summarised in Table 2.

**Table 2:** HCV genotype and subtype results according to the genotyping method used (*n*).

Group	GT II (5′NC, NS5B)	GT *Plus* (core)	NS5B sequencing	Core sequencing	LiPA (5′NC, core)
Group 1	1 *(152)*	1a *(18)*	1a *(16)*	1a *(16)*	—
			—	—	1a (*1*)
			1b *(1)*	1b *(1)*	—
		1b *(117)*	1b *(72)*	1b *(72)*	—
			1b *(1)*	failed *(1)*	—
			1b (1)	—	—
			—	—	1b (*42*)
			1 g *(1)*	1 g *(1)*	—
		Not detected (17)	1b *(12)*	1b *(12)*	—
			1d *(1)*	—	—
			1e *(1)*	1e *(1)*	—
			1 g *(1)*	1 g *(1)*	—
			1 h *(1)*	1 h *(1)*	—
			failed (*1*)	failed (*1*)	1b (*1*)
	1, 2 *(1)*	1b *(1)*	2i *(1)*	1b *(1)*	—
	1, 3 *(2)*	1b *(2)*	1b *(1)*	1b *(1)*	—
			—	—	1b (1)
	1, 4 *(5)*	1a *(2)*	1a (*2*)	1a *(2)*	—
		1b *(2)*	1b *(2)*	1b *(2)*	—
		Not detected (*1*)	4m *(1)*	—	—
Group 2	1a *(16)*	1a *(15)*	—	—	—
		1a, 1b *(1)*	1a *(1)*	1a *(1)*	—
	1b *(13)*	1b *(11)*	—	—	—
		Not detected *(2)*	1b *(2)*	1b *(2)*	—
	1b, 2 *(1)*	Not detected (*1*)	—	—	2a/2c *(1)*
	1b, 3 *(1)*	Not detected *(1*)	3a *(1*)	1b *(1)*	—
	1b, 3, 4 *(1)*	Not detected (*1*)	1b *(1)*	1b *(1)*	—
	1a, 1b *(6)*	1a, 1b *(1)*	1b *(1)*	1b (*1)*	—
		1a *(2)*	1a *(2)*	1a *(2)*	—
		1b *(3)*	1b *(3*)	1b *(3)*	—

GT II, Abbott RealTi*me* HCV Genotype II assay; GT *Plus*, Abbott HCV Genotype *Plus* RUO assay; LiPA, VERSANT HCV Genotype 2.0 assay; NGS, next-generation sequencing.

### Ability of the GT *Plus* assay for subtyping genotype 1 samples with no subtype assigned by the GT II assay

**Figure 2:**
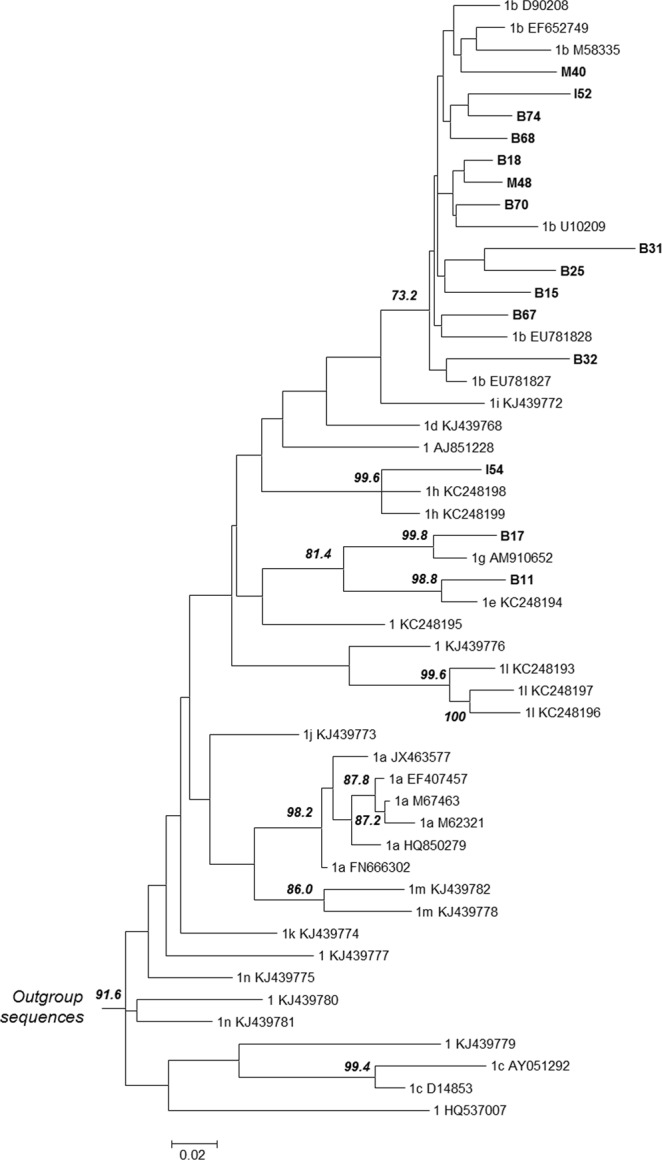
Maximum likelihood phylogenetic subtree of the genotype 1 core region (which includes the region targeted by the GT *Plus* assay) for samples belonging to Group 1 with a “not detected” result by the GT *Plus* assay. Among the 18 “not detected” samples, sequencing of the core region failed for sample I51 and could not be performed for samples I23 and I45 (not available). Sequences available in the Los Alamos National Library HCV sequence database (http://hcv.lanl.gov/content/index) and International Committee on Taxonomy of Viruses (ICTV) database^3^ were used as reference sequences. The General time-reversible substitution model was used (proportion of invariable sites: 0.394, gamma shape parameter: 0.694). Nodes supported with a bootstrap value > 70% (500 replicates) are indicated. Sequences from the “not detected” samples are indicated with their ID in boldface. The bar represents substitutions per nucleotide position. B, Badalona; I, Israel; M, Málaga. Outgroup sequences include the other HCV genotypes.

Among the 160 samples in Group 1, the GT *Plus* assay resolved the subtype in 142 cases (88.75%; 1a in 20 cases and 1b in 122 cases), while in the remaining 18 cases (11.25%) a “not detected” result was obtained. For the latter, the reference method based on the NS5B and core sequencing and phylogenetic analysis was used for HCV classification. Five out of 18 “not detected” cases correctly showed a “not detected” result as per assay design since sequencing identified genotype 1 subtypes other than 1a or 1b in four cases (subtypes 1d, 1e, 1g, and 1h) and a genotype 4m in the one case which previously was genotyped as HCV genotype 1 + 4 by the GT II assay. Another 12 cases were classified as 1b; evaluation of the sequences revealed the presence of mismatches in the core region in which the 1b-specific probes or primers of the GT *Plus* assay anneal. No specific cluster was observed in the phylogenetic tree of these “not detected” core sequences (Fig. 2), which include the region targeted by the GT *Plus* assay. In the remaining “not detected” case, sequencing failed in both regions due to low viral load (3.2 Log (IU/mL)) and LiPA was used for comparison to the GT *Plus* assay, which detected a subtype 1b. In samples with a “not detected” result, viral loads ranged from 3.2 to 6.9 Log (IU/mL) and, thus, were above the lower limit of detection of the GT *Plus* assay. Overall, among the samples with genotype 1 results without subtype assigned by GT II, the proportion of HCV subtype 1b isolates identified by sequencing or LiPA that could not be subtyped by the GT *Plus* assay was 9.6% (13/135) but varied geographically: 22.5% (9/40) in North-eastern Spain, 4.1% (2/49) in Southern Spain, and 4.3% (2/46) in Israel. Similarly, the proportion of non-1a non-1b subtypes, which are not covered by the GT *Plus* assay design, also varied geographically (1.8% in Spain and 4.0% in Israel), albeit the overall percentage was low.

### Concordance at the subtype level of the GT *Plus* assay with the reference method or LiPA in genotype 1 samples with no subtype assigned by the GT II assay

Among the 160 samples of Group 1 with no subtype assigned by the GT II assay, 116 samples underwent sequencing using the reference method, while remaining samples without sequencing results were compared to LiPA.

NS5B amplification and sequencing was successful in 115/116 samples (99.1%), and core sequencing in 111/113 samples (98.2%). Furthermore, HCV classification at subtype level based on the phylogenetic analysis of NS5B and core sequences agreed between the two target regions in all samples (Table 2) except for two, which were subjected to NGS (see below).

In those cases in which the GT *Plus* assay assigned a genotype 1 subtype (*n* = 98), the concordance between this subtype call and the reference method was 18/19 for subtype 1a (94.7%; one sample was classified as subtype 1b by sequencing), and 78/79 for subtype 1b (98.7%; one sample was classified as subtype 1g by sequencing). Thus, the overall agreement between the GT *Plus* assay and the reference method was 98.0% (96/98).

For the remaining 44 of 160 samples, the GT *Plus* assay assigned the subtype in all cases, and the LiPA assay was used as comparator to the GT *Plus* assay (Israel). The concordance between the genotype 1 subtypes identified by the GT *Plus* assay and by the LiPA assay, respectively, was 100% for both subtype 1a (1/1) and subtype 1b (43/43), respectively.

Overall, the accuracy of subtype 1a or 1b assignment by the GT *Plus* assay in comparison to either sequencing or LiPA was 140/142 (98.6%); i.e. 19/20 (95%) for subtype 1a and 121/122 (99.2%) for subtype 1b.

### Concordance at the subtype level between the GT *Plus* assay and the GT II assay in genotype 1a and 1b samples

When testing the 38 genotype 1a and 1b samples of Group 2 using the GT *Plus* assay, a genotype 1 subtype was assigned in 33 cases (86.8%). The agreement between both assays at subtype level was 15/16 for subtype 1a (93.8%; one sample was classified as mixed 1a + 1b subtypes by GT *Plus*), 11/11 for subtype 1b (100%), and 1/6 for those detected as mixed 1a + 1b subtypes (16.7%; two samples were classified as 1a by GT *Plus* and three as 1b). The GT *Plus* result was “not detected” in the remaining samples (*n* = 5): four of them were 1b by sequencing and the other one, initially detected as 1b + 2 by the GT II assay, was genotype 2 by LiPA. The overall concordance for single infections with either subtype 1a or 1b between both Abbott assays was 96.3% (26/27 samples). If samples with mixed 1a + 1b subtypes by the GT II assay were included and only considered as concordant if both GT II and GT *Plus* assays detected the mixed subtypes, the overall concordance was 81.8% (27/33 samples).

### Assessment of mixed infections

Seven 1a + 1b mixed subtypes were detected either with the GT *Plus* assay (*n* = 2) and/or the GT II assay (*n* = 6). These mixed subtypes were assessed by Sanger (core and NS5B regions) and NGS (NS5B region) but none of these methods confirmed a presence of mixed subtypes (Table 3). Evaluation of the generated sequences revealed perfect matches in the probe regions of the GT II or GT *Plus* assay for the subtypes that were confirmed by sequencing while mismatches were observed for the second subtypes.

**Table 3:** Sequencing results in samples with mixed HCV 1a + 1b subtypes as detected by either the GT II and/or the GT *Plus* assay.

Sample ID	Viral load (LogIU/mL)	HCV subtype result according to the genotyping method used						
		GT II (5′NC and NS5B)*	GT *Plus* (core)*	NS5B sequencing	Core sequencing	NS5B NGS	NS5B NGS % reads	NS5B NGS No. reads
B44	6.80	1a	1a, 1b	1a	1a	1a	99.3**	28,125
M56	6.94	1a, 1b	1a, 1b	1b	1b	1b	100	218,475
M34	7.93	1a, 1b	1b	1b	1b	1b	100	13,623
M52	6.52	1a, 1b	1b	1b	1b	1b	100	97,539
M53	Unknown	1a, 1b	1b	1b	1b	1b	100	56,280
M57	6.93	1a, 1b	1a	1a	1a	1a	100	70,548
M68	7.11	1a, 1b	1a	1a	1a	1a	99.2**	62,310

GT II, Abbott RealTi*me* HCV Genotype II assay (Abbott Molecular Inc.); GT *Plus*, HCV Genotype *Plus* RUO assay (Abbott Molecular Inc.); NGS, next-generation sequencing; B, Badalona; M, Málaga.

*In case of mixed subtype results, perfect match in the probe target region for the subtype that was also identified by sequencing while the other subtype showed mismatches.

**100% was not achieved because residual sequences appeared which were filtered and discarded as they were below 1%.

Eleven mixed genotypes were detected by the GT II assay, eight in Group 1 and three in Group 2. The GT *Plus* assay results in these samples were as follows (Table 4): (i) the genotype 1 subtype was resolved in seven specimens and results were confirmed either by the reference method in the targeted core region (*n* = 6; two 1a and four 1b) or by LiPA (*n* = 1; 1b); (ii) the GT *Plus* correctly showed a “not detected” result in one sample since sequencing revealed the presence of genotype 4m; (iii) similarly, a “not detected” result was obtained in one sample from Israel (I35) showing a result pattern across the assays that suggested a recombinant St. Petersburg variant consisting of genotype 2k in the 5’NC and core regions and 1b in NS5B^31^ (GT II: genotype 2 in the 5′NC and 1b in the NS5B regions; LiPA: 2a/2c in 5’NC and core regions; and GT *Plus*: “not detected” in the core region since genotype 2 is not detected per assay design) but, unfortunately, there was no sample material left to perform sequencing for confirmation; and (iv) two samples with a “not detected” result of GT *Plus* turned out to be genotype 1b by sequencing of the targeted core region. Subsequently, all these samples with mixed genotypes were subjected to NGS, except for the three cases from Israel due to lack of residual sample material (Table 4). In one case (M49), NGS detected mixed 1a + 1b subtypes that had not been identified by any of the previous assays. The last two specimens in Table 4 (M51 and M54) showed discordant results at the genotype level between core and NS5B Sanger sequencing and, thus, NGS was performed targeting the 5′NC-core region in addition the NS5B to identify the cause of these discordant results. However, NGS did not detect any mixed genotypes but identified genotype 2i for specimen M51 and genotype 3a for specimen M54 in both target regions, respectively. Thus, NGS ruled out recombination between different genotypes as the cause of these rare discordant results. Nevertheless, since genotype 1 primers had been used to amplify the core region during Sanger sequencing and pangenotypic ones during NGS, it could be possible that the former ones had been able to amplify a minority population of 1b genomes that were below the NGS cut-off, i.e. Sanger sequencing of the core region could have revealed the presence of a 1b subpopulation besides genotype 2i for patient M51 and 3a for M54 being the dominant population. Therefore, taking all data into account for specimens M51 and M54, we consider mixed genotypes, with subtype 1b below the NGS cut-off, as the most probable result. Overall, NS5B NGS results confirmed those obtained by Sanger sequencing in 14/15 cases (93.3%) with suspected mixed subtypes or genotypes by Abbott assays.

**Table 4:** Sequencing results in samples with mixed genotypes detected by the GT II assay.

Sample ID	Viral load (LogIU/mL)	HCV subtype result according to the genotyping method used							
		GT II (5′NC and NS5B)	LiPA (5′NC, core)	GT *Plus* (core)	NS5B sequencing	Core sequencing	NS5B NGS	NS5B NGS % reads	NS5B NGS No. reads
B1	5.08	1, 4	—	1a	1a	1a	1a	99.4**	27,971
B29	5.74	1, 4	—	1a	1a	1a	1a	100	44,412
M49	5.19	1, 4	—	1b	1b	1b	1b 1a	86.6 13.4	23,436 3,643
M67	7.43	1, 4	—	1b	1b	1b	1b	100	79,335
I23	5.38	1, 4	—	Not detected	4m	—	—	—	—
M35	6.60	1, 3	—	1b	1b	1b	1b	98.8**	78,924
I10	4.60	1, 3	1b	1b	—	—	—	—	—
B64	5.30	1b, 3, 4	—	Not detected	1b	1b	1b	100	48,744
I35*	6.32	1b, 2	2a/2c	Not detected	—	—	—	—	—
M51	5.92	1, 2	—	1b	2i	1b	1b	100	48,744
M54	6.73	1b, 3	—	Not detected	3a	1b	3a	99.3**	89,058

GT II, Abbott RealTi*me* HCV Genotype II assay (Abbott Molecular Inc.); GT *Plus*, HCV Genotype *Plus* RUO assay (Abbott Molecular Inc.); LiPA, VERSANT HCV Genotype 2.0 assay (Siemens Healthcare GmbH); —, not tested; B, Badalona; I, Israel; M, Málaga.

*Combination of results of all 3 assays is in agreement with a recombinant 2k/1b (St. Petersburg) variant, however, no material was left for sequencing to confirm.

**100% was not achieved because residual sequences appeared which were filtered and discarded, being each of them below 1%.

## Discussion

In the era of direct-acting antivirals against HCV infection, an accurate genotyping as well as a reliable discrimination between subtype 1a and 1b remains clinically and epidemiologically relevant^9,10,32^. However, commercial genotyping assays may result in a low percentage of HCV-1 results with no subtype assigned^11–14,18–21^. The GT II assay, which targets the NS5B region for identifying subtypes 1a and 1b, may fail to resolve the subtype in about 5–12% of HCV-1 specimens^11,13,14,33^. However, a recent GT II software update has reduced the number of genotype samples without subtype assignment (4% in Herman *et al*.^21^). An inability of the GT II assay to identify the genotype 1 subtype may be due to (i) the presence of genetic variability in the NS5B region in which the probes specific for subtype 1a or 1b detection anneal (majority in the present study), (ii) the presence of rare HCV-1 subtypes (four cases observed), or (iii) the presence of certain genotype 6 subtypes in which the 5′NC region targeted by the genotype 1-specific probe is identical to some 1b subtypes^11,13,33^ (none observed in our study).

We evaluated the performance and accuracy of the novel GT *Plus* assay in resolving HCV-1 specimens not subtyped by the GT II assay (Group 1) in comparison with the reference method or with LiPA. While three other evaluations of the GT *Plus* assay have previously been published^11,33,34^, the present study investigated HCV-1 specimens including the largest collection of very challenging clinical specimens with ambiguous HCV-1 results by GT II from three different geographical regions that was ever tested with the GT *Plus* assay and subsequently evaluated by a reference method for confirmation. Additionally, it was the first study in generating full core region sequences that shed some light into the reasons why this assay may not assign an HCV-1 subtype in rare cases. This HCV sequencing information will also be useful for other manufacturers of core-based genotyping assays.

Herein, the GT *Plus* assay resolved the subtype in 88.8% (142/160) of Group 1 cases, while a “not detected” result was obtained for the remaining 18 specimens. Since the GT *Plus* assay is designed to identify HCV subtypes 1a and 1b as well as genotype 6, three main reasons may be pivotal if the GT *Plus* assay provides a “not detected” result for genotype 1 samples without subtype by the GT II assay. Firstly, HCV-1 subtypes other than 1a and 1b may be present which are not detected by the GT *Plus* assay due to its genotype coverage (i.e. 1a, 1b, 6) per assay design. In the current study, 4/18 (22%) “not detected” samples were classified as 1d, 1e, 1g, and 1h by sequencing, while the proportion of non-1a non-1b subtypes was higher in the study by Mokhtari *et al*. in France (25/32, 78.1%)^11^. Secondly, another potential reason is the presence of mismatches in the core region targeted by HCV 1a-, 1b- and 6-specific primers and probes used by the GT *Plus* assay. In the present study, evaluation of the core sequences confirmed this in 12/18 (66.7%) samples that were “not detected” by the GT *Plus* but classified as subtype 1b by sequencing (for another sample sequencing failed). This proportion was higher than in the studies by Mokhtari *et al*. (9.4%, 3 out of 32 cases, which were also 1b)^11^ and Mallory *et al*. (7.1%, 1 out of 14 samples, being 1a)^34^. Thirdly, in rare cases, a sample can be incorrectly genotyped as HCV-1 by GT II, and consequently, the GT *Plus* returns a “not detected” result. Indeed, we observed a “not detected” result in a sample originally identified as mixed 1 + 4 genotypes by the GT II assay that turned out to be subtype 4m by sequencing. This was also observed by Mallory *et al*., who reported a “not detected” result in a sample previously identified as HCV-1 with no subtype assigned by the GT II assay and classified as genotype 4 by sequencing^34^. The polymorphisms present in specific HCV isolates circulating in different geographical areas (genetic diversity both among HCV-1 subtypes and within subtype 1b) influenced the proportion of “not detected” results by GT *Plus* in genotype 1 samples without subtype by GT II: it increased from 3.5% (2/57) in Southern Spain to 10% (5/50; including the genotype 4 sample) in Israel, and up to 20.8% (11/53) in North-eastern Spain (overall 18/160, 11.3%). However, it was lower than previously reported: 32/98 (32.7%) in France^11^, and 7/14 (50.0%) in the USA^34^.

The accurate recognition of subtypes 1a and 1b represents a common and difficult challenge to commercial assays. In the present study, the GT *Plus* assay demonstrated to be highly reliable in assigning 1a and 1b subtypes in specimens initially not subtyped by the GT II assay (Group 1), as the proportion of calls by the GT *Plus* assay that were confirmed by sequencing was very high (98.0%; 96 out of 98 samples). This proportion was higher than that observed in Mokhtari’s study (84.3%; 43 out of 51 samples)^11^, but similar to that reported by Mallory *et al*. (97.1%; 168 out of 173 samples)^34^. In agreement with the latter study, we suggest a non-specific reactivity as the most probable cause for the rare discordant results observed in the present study; one subtype 1b misclassified by the GT *Plus* as subtype 1a, and one subtype 1g misclassified as subtype 1b. When we included LiPA results for those samples in which sequencing was not done, overall agreement at the subtype level (with either LiPA or sequencing) even slightly increased to 98.6% (140/142), as the agreement at HCV-1 subtype calling between the GT *Plus* and the LiPA assays was 100% (all being 1b except one 1a case). The LiPA assay also relies on the core region for the identification of subtypes 1a and 1b, and genotype 6. However, it may fail to correctly identify 1a and 1b subtypes in a variable percentage of cases (5–14%)^15–18^, and tends to misclassify genotype 6 as genotype 1^16,23^. While the latter is unlikely in Spain and Israel, we have relied on the LiPA assay for assessment of GT *Plus* HCV-1 subtype calls in 31% of the cases. As this fact may be a limitation of our study, we have analysed the concordance between the GT *Plus* and LiPA assays separately from the concordance between the GT *Plus* assay and sequencing.

The identification of mixed infections represents another challenge for current commercially available assays based on real-time PCR or line-probe hybridisation^13,16,19,21,35,36^. Mixed infections have been reported particularly in those patients exposed to multiple HCV infections such as people who inject drugs^37^. It has been suggested that NGS may improve the detection of minor genotypes/subtypes in mixed infections; however, NGS assays including the one used herein^38^ usually require comparably high viral loads, while real-time subtype-specific nested reverse transcriptase PCR can detect virus at low concentrations^37^. It has previously been suggested that the GT II assay is able to reliably detect mixed infections if the minor genotype/subtype represents at least 20–30% of the circulating variants (similarly to Sanger sequencing)^13,35^. Furthermore, it is able to genotype at viral loads of 500 IU/mL and below depending on the genotype^39^. In our study, NGS of the NS5B region did not detect mixed 1a + 1b subtypes as previously identified by either the GT *Plus* (*n* = 1), the GT II (*n* = 5) or both (*n* = 1) assays. Alternatively, the detection of the two mixed 1a + 1b subtypes by the GT *Plus* assay could have been due to cross-reactivity between both subtypes. The latter is supported by our evaluation of the sequences that revealed no mismatches in the probe target region for the subtypes confirmed by sequencing while there were mismatches observed for the other subtypes, respectively. On the other hand, in a sample classified as mixed 1 + 4 genotypes without genotype 1 subtype assignment by GT II, only NS5B NGS identified mixed 1a + 1b subtypes while GT *Plus* and Sanger sequencing of both NS5B and core regions concordantly classified it as 1b only. Lastly, in order to further characterise two specimens with discordant results between core and NS5B Sanger sequencing (1b/2i and 1b/3a), two distant genomic regions of the virus (5′NC-core and NS5B) were subjected to NGS. While the NGS assay did not confirm the presence of mixed genotypes in these specimens, the evidence gathered from Sanger sequencing and real-time PCR assays nevertheless points to the presence of both genotypes, respectively. These few examples show that even the application of gold standard methods may lead to discordant results and cannot unambiguously resolve the HCV genotype of some specifically challenging samples. Hence, more data is required to establish the lowest viral load that can be reliably detected by NGS for minor types^37^.

Finally, when focusing on the results from North-eastern Spain (Germans Trias i Pujol University Hospital), 5.7% of all genotypes 1 from 2009 to 2016 (76 out of 1322) were not classified at the subtype level by the GT II assay. Among them, 53/76 left-over plasma samples were available and analysed with the GT *Plus* assay in this study. In 42/53 (79.2%) the subtype was successfully resolved. Thus, we can estimate that the use of the GT *Plus* assay as a reflex test after the GT II assay would have decreased the overall ratio of HCV-1 samples in need of subtyping by the reference method from 5.7% down to 1.1%, which translates into a successful resolution of 98.9% of all HCV-1 samples when the two Abbott genotyping assays are combined. This corresponds well to the recently reported significant reduction of genotype 1 cases without subtype assignment down to 0.4%^33^ and to the increase of the successful genotyping rate to >99%^11^.

In conclusion, in the largest study focused on HCV-1 specimens not subtyped by the GT II assay, the GT *Plus* assay was able to assign the subtypes 1a and 1b in the majority of cases (88.8%), and subtype calling was highly reliable (98.6%) in comparison with sequencing or LiPA. The GT *Plus* assay is fully automated and can easily and rapidly be performed in the laboratory. Consequently, combined application of the GT II assay followed by the GT *Plus* assay using the Abbott *m*2000 platform represented an adequate approach which substantially reduced the number of ambiguous genotype 1 samples requiring subtyping by alternative methods down to 1.1%.

## Supplementary information

Table S1

## Data Availability

Protocols used are specified or referenced in the manuscript. NS5B and core sequences obtained by Sanger sequencing were deposited in the EMBL sequence database (http://www.ebi.ac.uk/embl). All commercial assays were used following the instructions of the manufacturers. The Abbott HCV GT *Plus* RUO assay is not commercially available. Data other than proprietary information can be provided on request.
