# Supplementary material for: Reliable resolution of ambiguous hepatitis C virus genotype 1 results with the Abbott HCV Genotype Plus RUO assay
Source: Sci Rep. 2019 Mar 6;9:3678. doi: 10.1038/s41598-019-40099-3 (PMC6403303; doi:10.1038/s41598-019-40099-3)
Supplement: Supplementary file 1 — Table S1 [file 41598_2019_40099_MOESM1_ESM.docx]

***Supplementary Information***

**Reliable resolution of ambiguous hepatitis C virus genotype 1 results with the Abbott HCV Genotype *Plus* RUO assay**

V. Saludes^1,2,3^, A. Antuori^1^, B. Reinhardt^4^, I. Viciana^5^, E. Clavijo^5^, L. Schreiber^6^, M. Tenenbaum^6^, F. Rodriguez-Frias^7,8^, J. Quer^8,9^, L. Matas^1,3^, and E. Martró^1,2,3*^.

^1^ Microbiology Service, Germans Trias i Pujol University Hospital, Genetics and Microbiology Department, Universitat Autònoma de Barcelona, Badalona.

^2^ Germans Trias i Pujol Research Institute (IGTP), Can Ruti Campus, Badalona, Spain

^3^ Centro de Investigación Biomédica en Red en Epidemiología y Salud Pública (CIBERESP), Instituto de Salud Carlos III, Madrid, Spain

^4^ Abbott GmbH & Co. KG, Wiesbaden, Germany

^5^ Microbiology Service, Hospital Virgen de la Victoria, Málaga, Spain

^6^ Maccabi Mega-Lab, Rehovot, Israel

^7^ Liver Pathology Lab, Biochemistry and Miocrobiology services, University Hospital Vall d'Hebron, Barcelona, Spain

^8^ Centro de Investigación Biomédica en Red (CIBER) de Enfermedades Hepáticas y Digestivas (CIBERehd), Instituto de Salud Carlos III, Madrid, Spain

^9^ Liver Unit, Internal Medicine, Lab. Malalties Hepàtiques, Vall d’Hebron Institute of Research (VHIR-HUVH), Barcelona, Spain

*Corresponding author: Elisa Martró. Microbiology Service, Germans Trias i Pujol University Hospital, Crta. del Canyet s/n, 08916 Badalona (Barcelona), Spain. E-mail: [emartro@igtp.cat](mailto:emartro@igtp.cat); Phone: +34 934978894. Fax: +34 934978895.

**Table S1. Primers and thermal cycler conditions used for the amplification of the core region.**

| **PCR**  **strategy** | **PCR round** | **Primer** | **Sequence (5’-3’)** | **H77 (AF009606)**  **positions*** | **Sense** | **Primer**  **reference** | **Thermal cycler conditions** |
| --- | --- | --- | --- | --- | --- | --- | --- |
| First | Primary | Cg1 | GCCATRGTGGTCTGCGGAAC | 137–156 | F | 1 | 1cycle (94°C, 2 min),  35 cycles (94°C, 1 min; 55°C, 2 min; 72°C, 3 min),  1cycle (72°C, 7 min). |
|  |  | Ca | GTTGGAGCAGTCGTTCGTRA | 949–968 | R | 1 |  |
| Second | Primary | Cg1 | GCCATRGTGGTCTGCGGAAC | 137–156 | F | 1 | 1 cycle (95°C, 2 min),  5 cycles (95°C, 1 min; 60°C, 30 s; 72°C, 1 min)  15 cycles (95°C, 1 min; 55°C, 30 s; 72°C, 1 min),  20 cycles (95°C, 1 min; 52°C, 30 s; 72°C, 1 min),  1cycle (72°C, 5 min). |
|  |  | Ca | GTTGGAGCAGTCGTTCGTRA | 949–968 | R | 1 |  |
|  |  | Core-1a-Rv-1 | acratrctcgarttrgggcart | 958–979 | R | - |  |
|  | Secondary | Cg1 | GCCATRGTGGTCTGCGGAAC | 137–156 | F | 1 |  |
|  |  | Ca | GTTGGAGCAGTCGTTCGTRA | 949–968 | R | 1 |  |
|  |  | Core-1a-Rv-2 | ttrgggcartcrttggtgacrt | 946–967 | R | - |  |
| Third | Primary | Core_F1 | CTAGCCATGGCGTTAGTATG | 79–98 | F | 2 | 1cycle (95°C, 2 min),  35 cycles (95°C, 1 min; 48.8°C, 2 min; 72°C, 3 min),  1cycle (72°C, 7 min). |
|  |  | CEi_r | TTCATCATCATRTCCCANGCCAT | 1293–1315 | R | 3 |  |
|  | Secondary | Core_F | ACTGCCTGATAGGGTGCTTGCGA | 288–310 | F | 4 | 1cycle (95°C, 2 min),  35 cycles (95°C, 1 min; 60°C, 2.5 min; 72°C, 3 min),  1cycle (72°C, 7 min). |
|  |  | CEi_r | TTCATCATCATRTCCCANGCCAT | 1293–1315 | R | 3 |  |

F, forward. R, reverse.

**References Table S1**

1. Saludes, V. *et al.* Baseline prediction of combination therapy outcome in hepatitis C virus 1b infected patients by discriminant analysis using viral and host factors. *PLoS One* **5**, e14132; doi: 10.1371/journal.pone.0014132. (2010).

2. Suzuki, F. *et al.* Case report: Clinical and virological analyses of a patient positive for hepatitis C virus-RNA by branched DNA assay but negative for anti-hepatitis C virus antibodies. *J. Gastroenterol. Hepatol.* **12**, 869–873 (1997).

3. Calado, R. A. *et al.* Hepatitis C virus subtypes circulating among intravenous drug users in Lisbon, Portugal. *J. Med. Virol.* **83**, 608–615 (2011).

4. Mallory, M. A., Lucic, D. X., Sears, M. T., Cloherty, G. A. & Hillyard, D. R. Evaluation of the Abbott realtime HCV genotype II RUO (GT II) assay with reference to 5’UTR, core and NS5B sequencing. *J. Clin. Virol.* **60**, 22–26 (2014).
